# Identifying piRNA targets on mRNAs in *C. elegans* using a deep multi-head attention network

**DOI:** 10.1186/s12859-021-04428-6

**Published:** 2021-10-16

**Authors:** Tzu-Hsien Yang, Sheng-Cian Shiue, Kuan-Yu Chen, Yan-Yuan Tseng, Wei-Sheng Wu

**Affiliations:** 1 Department of Information Management, National University of Kaohsiung, Kaohsiung, Taiwan; 2 Department of Electrical Engineering, National Cheng Kung University, Tainan, Taiwan; 3 Center for Molecular Medicine and Genetics, Wayne State University, School of Medicine, Detroit, MI USA

**Keywords:** piRNA, CLASH, Deep learning, Multi-head attention

## Abstract

**Background:**

Piwi-interacting RNAs (piRNAs) are the small non-coding RNAs (ncRNAs) that silence genomic transposable elements. And researchers found out that piRNA also regulates various endogenous transcripts. However, there is no systematic understanding of the piRNA binding patterns and how piRNA targets genes. While various prediction methods have been developed for other similar ncRNAs (e.g., miRNAs), piRNA holds distinctive characteristics and requires its own computational model for binding target prediction.

**Results:**

Recently, transcriptome-wide piRNA binding events in *C. elegans* were probed by PRG-1 CLASH experiments. Based on the probed piRNA-messenger RNAs (mRNAs) binding pairs, in this research, we devised the first deep learning architecture based on multi-head attention to computationally identify piRNA targeting mRNA sites. In the devised deep network, the given piRNA and mRNA segment sequences are first one-hot encoded and undergo a combined operation of convolution and squeezing-extraction to unravel motif patterns. And we incorporate a novel multi-head attention sub-network to extract the hidden piRNA binding rules that can simulate the biological piRNA target recognition process. Finally, the true piRNA–mRNA binding pairs are identified by a deep fully connected sub-network. Our model obtains a supreme discriminatory power of AUC $=$ 93.3% on an independent test set and successfully extracts the verified binding pattern of a synthetic piRNA. These results demonstrated that the devised model achieves high prediction performance and suggests testable potential biological piRNA binding rules.

**Conclusions:**

In this research, we developed the first deep learning method to identify piRNA targeting sites on *C. elegans* mRNAs. And the developed deep learning method is demonstrated to be of high accuracy and can provide biological insights into piRNA–mRNA binding patterns. The piRNA binding target identification network can be downloaded from http://cosbi2.ee.ncku.edu.tw/data_download/piRNA_mRNA_binding.

## Background

Piwi-interacting RNAs (piRNAs) are small non-coding RNA molecules found in animals that help protect genome integrity by silencing transposons [1]. Studies reported that they could silence numerous transposable elements through the interaction with the PIWI-clade Argonautes [2]. Since then, numerous piRNAs produced in animal genomes are recognized. However, most known piRNAs do not match transposon sequences, from nematodes to mice [3, 4]. These results suggest the existence of additional piRNA targets and functions in cells. For example, researchers have found that piRNAs can target and regulate various endogenous mRNAs in mouse sperm production and in fly early embryo mRNA localization [5, 6]. Hence, understanding how piRNAs bind and regulate the expression of their mRNA targets is an imminent issue in animal gene transcriptional control.

While numerous efforts have been made to understand the binding targets of microRNAs (miRNAs), which is another primary class of small RNAs in cells, there are only few known results on the piRNA target sites. And the understanding of miRNA-mRNA interactions does not reasonably apply for piRNA since piRNA has its distinguishing characteristics: (1) piRNAs do not show any conservation signals in their primary nor secondary structures [7]; (2) piRNAs bind to Piwi-clade Argonautes while miRNAs work with Ago-clade Argonautes [8]; (3) piRNAs have different biogenesis mechanisms that do not depend on Dicer [9]. Recently, the piRNA targeting rules in *C. elegans* was refined by a piRNA reporter assay [10]. The results showed that piRNAs require near-perfect matching within a 2–7 nt-long seed region. However, unlike miRNAs, only a few mismatches outside the seed region can be found in piRNA targeting mRNA sites, and the first base does not get involved in piRNA targeting. These newly discovered phenomenons confirm the need for developing independent piRNA target investigation methods. Nevertheless, there is still no systematic way to assess the transcriptome-wide piRNA targeting sites due to the current limited understanding of the piRNA–mRNA binding mechanisms.

In silico methods can provide a first-hand understanding of how piRNAs target and silence endogenous genes other than transposons in cells [11]. A computational model that helps infer the potential regulatory functions and the binding mechanisms can be utilized to transcriptome-widely screen out possible binding target sites of piRNAs [12–14]. However, to our knowledge, there is only one such tool that helps computationally identify piRNA targets on mRNAs till now. In [15], an SVM method that relies on hand-crafted, position-derived, and Miwi CLIP-seq (cross-linking immunoprecipitation coupled with deep sequencing) derived features were developed for extracting piRNA targets in mouse mRNAs. Nevertheless, the manually crafted piRNA and mRNA features restrict the generalization of the methods to other animal species. Furthermore, in such machine learning-based algorithms, the generation of confident experimentally verified negative sets highly affects the false-positive rates of the trained models. The existing SVM-based method was trained on a small dataset with mutant expression-inferred negative samples instead of verified negative pairs. These properties may lead to high false positives and hinder the model application to other animal species.

In this research, the first deep learning framework for piRNA targeting mRNA site prediction was designed to overcome the aforementioned obstacles. We first automatically generated the motif features for the given piRNA sequences and mRNA segments using the convolutional filtering and squeezing-and-excitation blocks. And a novel multi-head attention sub-network is incorporated into the deep learning network architecture to extract piRNA binding rules that can simulate the cellular piRNA binding pattern recognition. These recognized binding patterns are then fed into a fully connected classification sub-network to distinguish real piRNA binding mRNA sites from random piRNA–mRNA pairs. To elude the false positive trap caused by unverified lower quality negative sets, we adopted the experimentally validated positive sets from the cross-linking, ligation, and sequencing of hybrids (CLASH) identified piRNA–mRNA binding signals and devised a novel procedure to obtain a confident validated negative set. Under random training/validation/test splits on the positive and the negative sets from the wild-type CLASH data, the devised network obtained a test AUC (area under the curve) of 95.7% and demonstrated good generalization from the validation stage to unknown test samples. We further showed that the multi-head attention incorporated in this network could boost the network performance to bring out 6.7% (93.3–86.6%) AUC improvement over the pure convolutional neural network (CNN) model on an independent CSR-1 depletion CLASH test set. Finally, we demonstrated the biological interpretability of our devised model via piRNA–mRNA pairs that were fully experimentally explored. The examples indicated that our model could convey cellular mechanisms through the incorporated novel multi-head attention operation. The devised network not only provides superior piRNA–mRNA binding event prediction but also suggests potential biological piRNA binding mechanisms. We believe that the devised deep learning network can help biologists discover unknown piRNA targeting mRNA sites and pave the way for understanding piRNA-induced gene silencing.

## Results

### Overview of the deep learning piRNA binding target identification process

**Fig. 1:**
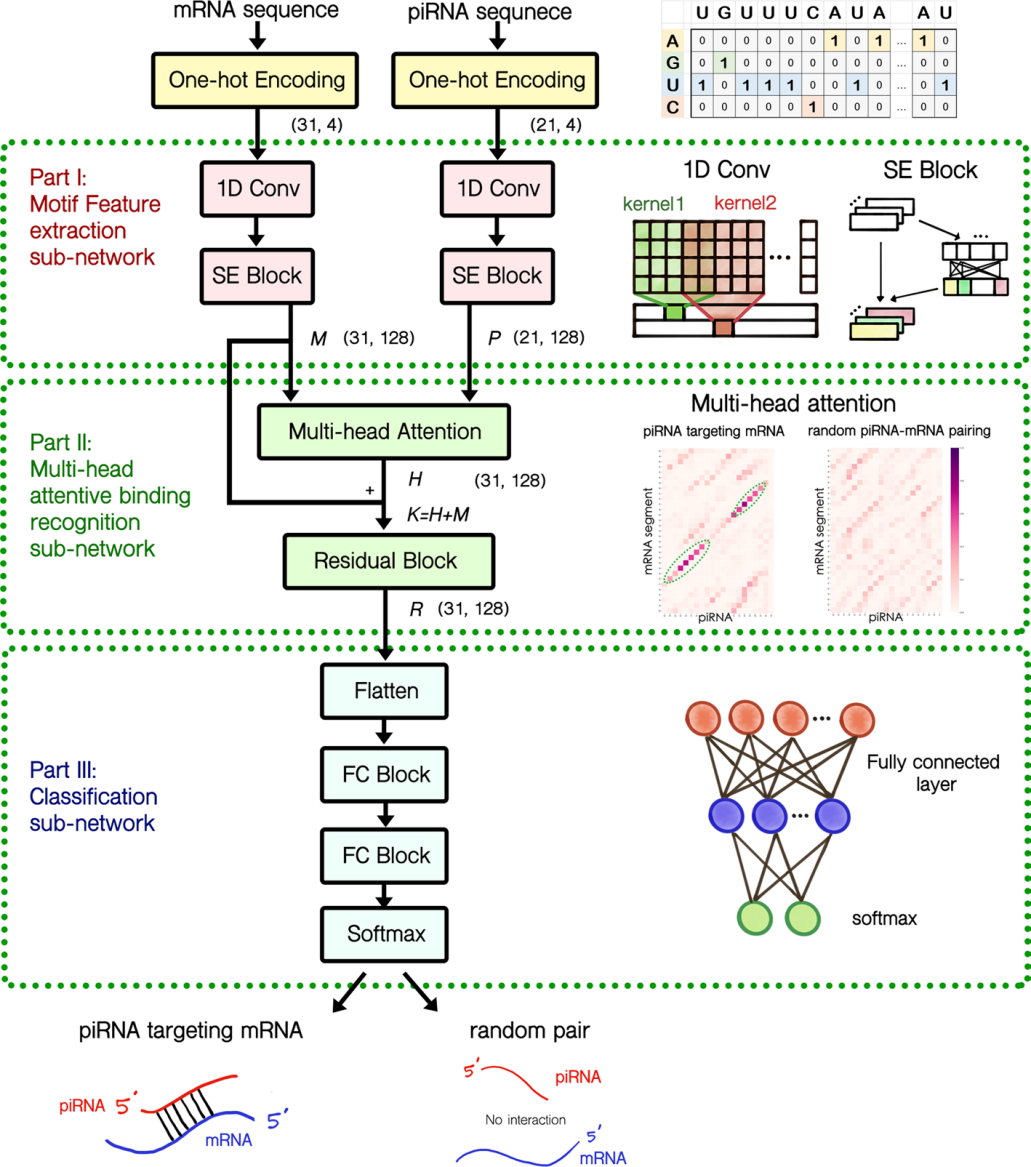
The overview of the devised deep multi-head attention network for identifying piRNA–mRNA binding events. The network can be divided into three different parts. First, the site-by-site motif features of the given piRNA and mRNA segment are extracted by convolution and squeezing-excitation operations. Then the piRNA binding rules are mimicked by the multi-head attention operation. Finally, the piRNA binding mRNA targets are identified using the attentive feature vectors. The pair (x, y) in the graph indicates the dimension of the operation output matrix

In this research, we designed a novel deep neural network architecture that automatically generates significant motif patterns that can help identify piRNA–mRNA binding interactions. It is now known that piRNAs bear special motif preferences, such as perfect match seed regions [10]. Hence it is beneficial to consider piRNA and its targeting mRNA segments simultaneously in understanding piRNA binding mechanisms. A novel mechanism based on multi-head attention techniques was incorporated into the devised network to achieve the piRNA binding preference recognition. The developed network takes a piRNA sequence and a potential targeting mRNA sequence segment as inputs and provides the results showing if the piRNA binds to the mRNA segment and potentially triggers transcriptional silencing. The devised network can be roughly divided into three sub-networks: the motif feature extraction sub-network, the multi-head attentive binding recognition sub-network, and the classification sub-network (see Fig. 1). Before input into the devised network, the piRNA and mRNA segment sequences are converted into matrices using nucleotide one-hot encoding. These one-hot encoded sequence matrices are then fed into the motif feature extraction sub-network to automatically extract substantial motif features from the sequence pattern (Fig. 1-Part I). The multi-head attention operation then aligns these piRNA and mRNA motif features (Fig. 1-Part II). In the multi-head attentive binding recognition sub-network, the multi-head attention operation simulates the binding recognition process for the piRNA–mRNA pair. Finally, the attentive vector of the piRNA–mRNA pairing is forwarded into the classification sub-network for piRNA binding target identification (Fig. 1-Part III). The detailed information of these three sub-networks is described in the “The devised deep learning piRNA binding target identification network” subsection. And the network structure is summarized in Table 1.

**Table 1:** The devised piRNA–mRNA binding deep learning network structure

Sub-Networks	piRNA feature	mRNA feature
I: Motif feature extraction sub-network	Conv (size $=$ 5) * 128	Conv (size $=$ 5) * 128
	Batch-normalization	Batch-normalization
	PReLU	PReLU
	SE block (**P**)	SE block (**M**)
II: Multi-head attentive binding recognition sub-network	16-head attention layer (output: **H)**	
	Add Residual (**K** = **H+M**)	
	Layer-normalization	
	FC layer (hidden layer size = 128*4)	
	PReLU	
	FC layer (hidden layer size = 128) (**L**)	
	Add Residual (**K+L**)	
	Layer-normalization	
III: Classification sub-network	Flatten	
	FC layer (hidden layer size = 32*31)	
	Batch-normalization	
	PReLU	
	FC layer (hidden layer size = 8*31)	
	Batch-normalization	
	PReLU	
	Softmax layer	

conv represents the 1D convolution operation, SE stands for squeezing-and-excitation operation, FC is abbreviated for fully connected, PReLU is the Parametric Rectified Linear Unit, and capital letters are used to denote the output matrix results

**Fig. 2:**
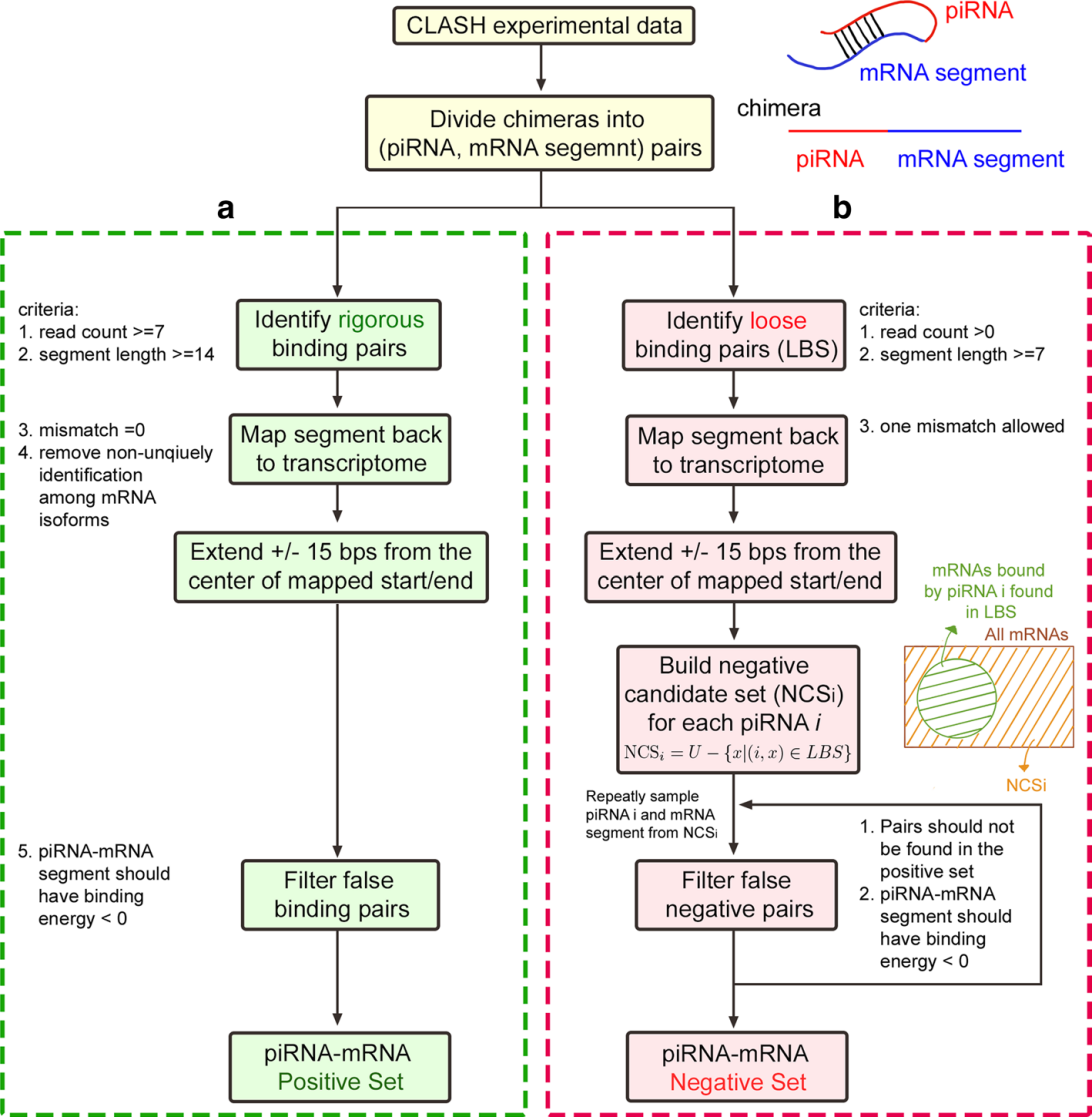
Steps for the positive and the negative piRNA–mRNA pairs preparation. **a** Rules for identifying real piRNA targeting mRNA sites. **b** Procedures to prepare the confident piRNA–mRNA non-associated pairs

Since the model prediction performance largely depends on the quality of the dataset preparation step, we collected the comprehensive wild-type *C. elegans* CLASH dataset from the work of Shen et al. [16] and further designed preprocessing steps to obtain the verified piRNA–mRNA positive binding set and verified negative piRNA–mRNA non-associated pair set. Currently, comprehensive CLASH experiments were only performed in *C. elegans*. Hence the worm species is selected to be used in this research. The wild-type piRNA–mRNA binding positive and negative sets can be downloaded from the URL indicated in the “Availability of data and materials” section. The preprocessing steps of the verified positive and negative sets are depicted in the following sub-sections (see Fig. 2).

#### The piRNA–mRNA positive binding set

**Fig. 3:**
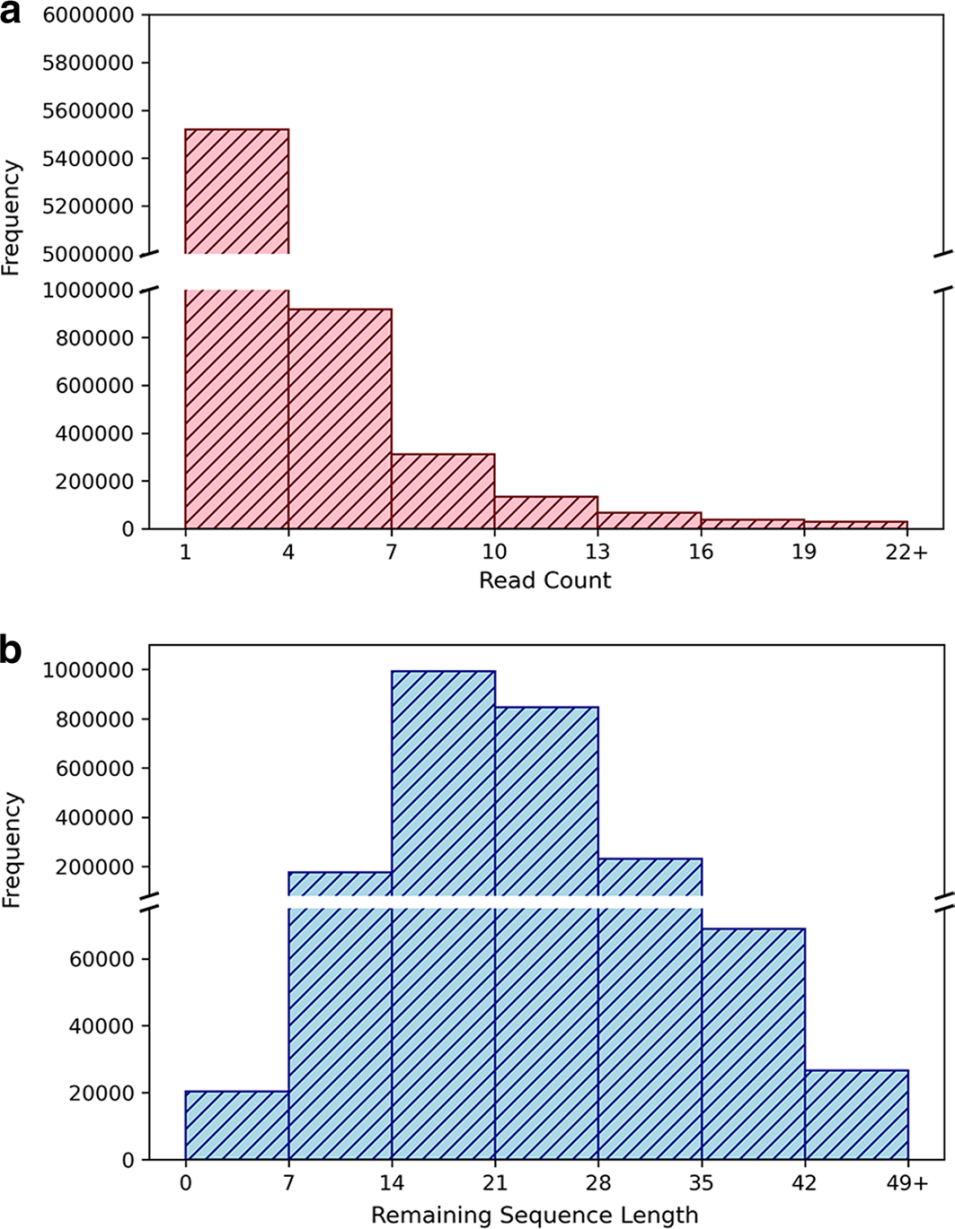
The basic sequencing information of the wild-type CLASH dataset. **a** The read count distribution of the raw chimera reads. **b** The distribution of the lengths of the remaining sequences, or the sequences that were probed to potentially hybridize with some piRNA

The positive set includes the real in vivo probed mRNA targets of known piRNAs. Chimeras sequenced from the CLASH experiments provide evidence for the interaction between piRNAs and their mRNA targets. First, we obtained the chimeric sequences with a perfect match of part of their segments to some known piRNA sequence (annotated by WormBase [17] WS275.PRJNA13758) using bowtie [18]. Then the remaining segment of a chimera (excluding the piRNA matching sequence) is regarded as the mRNA target of the matched piRNA [19]. We aligned the identified mRNA targets back to the *C. elegans* transcriptome (WormBase version WS275.PRJNA13758) using bowtie with default parameters. The center site of the mapped start and end transcript locations of the mRNA segment was denoted as the measured piRNA target site. Due to the RNase treatment in the CLASH protocols, the chimeras may suffer from RNA degradation. To recover the potential RNA degradation, 15 bps were extended upstream and downstream of the target site to be the final targeting mRNA segment. Since chimeras can be formed from random RNA ligations in CLASH-seq, we restricted the identified real rigorous piRNA–mRNA pairs to satisfy the following criteria (see Fig. 2a): (1) The read counts of the candidate chimeras should be at least 7. CLASH protocols often contain certain levels of background random ligated noise pairs. Hence, a minimum chimera read count threshold should be set up to eliminate the background random hybrid chimeras [20]. As shown in the read count distribution (Fig. 3a), chimera reads with read-count values less than 7 form frequency spikes and thus can not be easily distinguished from the background random hybrids. Therefore, a minimum read count of 7 was selected to exclude these possible random hybrids. (2) The remaining sequence of a chimera, excluding the piRNA matching sequence, should be at least 14 bps. Because hybrid ligation of short RNA fragments in CLASH protocols is limited [21], we need to enforce the minimum length of the remaining sequences, or the piRNA-bound mRNA segments, to ensure biologically meaningful pairs. From the length distribution of the remaining sequences (see Fig. 3b), 14 was decided since the mode value of the remaining sequence lengths happens around 14. The strict selection of 14 for the remaining sequence threshold can both remove most nonsense short hybrid ligated pairs and retain sufficient data for subsequent analysis. (3) There is no mismatch between the remaining sequence of a chimera with the aligned transcript segment. (4) When the binding target sequence of a specific piRNA is found multiple times among different mRNA transcript isoforms or within the same transcript, this piRNA–mRNA sequence pair is not included in the positive set to avoid adding a latent weight to this pair in the model training process. (5) The binding energy (calculated by RNAup [22]) between the piRNA and its targeting mRNA segment should be less than zero. By using the above five rigorous rules, a confident piRNA–mRNA binding positive pair set was formed. In total, 60,438 positive piRNA–mRNA binding pairs for 9397 confident targeting mRNA segments of 7126 known piRNAs were found using these rigorous rules.

#### The piRNA–mRNA negative pair set

We sought to gather the piRNA–mRNA pairs that do not form binding interactions in cells to build a confident negative set. For that purpose, a searching procedure was designed in this research to obtain a verified negative piRNA–mRNA random pair set (see Fig. 2b). The negative set includes the complementary pairs that are not in a loose piRNA–mRNA binding pair set (LBS). To obtain a strict negative dataset, LBS is required to be collected using loose filtering rules. First, the LBS was collected by the similar procedure of collecting the positive set, but with the following loose filtering rules: (1) the read counts of the candidate chimeras are larger than zero; (2) the remaining sequence of a chimera, excluding the piRNA matching sequence, is at least 7 bps; (3) 1 mismatch is allowed for the alignment of the remaining sequence of a chimera and the mRNA transcript. In the above three loose filtering rules, we allowed all possible observed chimera reads to be analyzed for encompassing as many potential week binding pairs as possible by setting the chimera read count threshold to be zero. Since hybrid ligation between two short RNA fragments is usually prohibited and biased in the CLASH protocols [21], we still took the relaxed remaining sequence length threshold of 7 (upper bound of the first bin, see Fig. 3b) to exclude the pure sequencing signal noises. We also relaxed the mismatch parameters in the alignment of the remaining sequences and the mRNA transcripts to include more possible week binding pairs. The threshold of one mismatch was selected to avoid the intensive computation caused by the data explosion when two mismatches were allowed in the bowtie alignment step. Although some non-binding piRNA–mRNA segment noise pairs may be included in LBS, the selected complementary pair that are not in LBS (i.e., the piRNA–mRNA negative pair) will thus be more strict and confident.

Based on the LBS, we obtained a negative candidate set ($\mathrm {NCS}_i$) for each of the known piRNA *i* included in the positive set using Eq. 1:

1 $$\begin{aligned} \mathrm {NCS}_i = U - \{ x | (i, x) \in LBS \}, \end{aligned}$$

where *U* denotes the set of all transcripts in *C. elegans* and (i, x) represents the loosely coupled piRNA *i* and target mRNA *x*. In this second step of the procedure, the known piRNAs refer to the 7126 piRNAs contained in the positive set. This restriction ensures that both binding and non-binding information for piRNA *i* is included in the ground truth data. Third, we randomly picked a known piRNA *p* and sampled a sequence of length 31 bps from some mRNA in $\mathrm {NCS}_p$. And finally, the selected piRNA–mRNA random associated pair is included in the negative set if (1) this pair is not aligned to any sequences of the positive set with maximum 2 mismatches and (2) the binding energy (calculated by RNAup [22]) between the piRNA and the randomly chosen mRNA segment should be less than zero to eliminate the trivial cases that the piRNA and mRNA cannot be physically paired. In total, 60,438 confident non-functional piRNA and mRNA segment associations were found using this negative set searching procedure.

### Model hyper-parameters and evaluation

We split the constructed positive and negative sets from the wild-type CLASH data into training/validation/test folds with a item ratio of 8:1:1 for 30 times. In the designed network, the model hyper-parameters were selected using the average performance of the split validation sets among the 30 iterations. The final best-chosen hyperparameters used in this research are the following: (1) Optimizer: Adam; (2) learning rate: 0.001 (3) learning scheduler: reduce-on-plateau, patience 5, factor 0.1, minimum learning rate 1e−6; (4) batch size: 512; (5) training epoch: 60, no early stopping; (6) activation function: parametric rectified linear unit; (7) dropout rate: 0.3 for dropout layers after the squeezing-and-excitation (SE) block and within the multi-head attentive binding recognition sub-network; 0.75 for dropout layers in the classification sub-network. Models of different structures were trained with Pytorch on NVIDIA RTX 2080 Ti GPUs.

The discriminatory power of the devised network is evaluated by calculating the percentage of correctly identified piRNA targeting mRNA sites and the percentage of correctly eliminated randomly associated piRNA–mRNA pairs. We can measure the model performance through metrics defined in the following equations [23]:

2 $$\begin{aligned} \mathrm {Sensitivity\, (recall)}= & {} \frac{\mathrm {TP}}{ \mathrm {TP} + \mathrm {FN} }, \ \mathrm {Specificity} = \frac{\mathrm {TN}}{ \mathrm {TN} + \mathrm {FP}} \end{aligned}$$

3 $$\begin{aligned} \mathrm {Accuracy}= & {} \frac{\mathrm {TP} + \mathrm {TN}}{ \mathrm {TP} + \mathrm {FP} + \mathrm {FN} + \mathrm {TN} } \end{aligned}$$

4 $$\begin{aligned} \mathrm {Precision}= & {} \frac{\mathrm {TP}}{ \mathrm {TP} + \mathrm {FP} }, \ \mathrm {F1} = 2 * \frac{\mathrm {precision} * \mathrm {recall}}{ \mathrm {precision} + \mathrm {recall} }, \end{aligned}$$

where TP represents the number of correctly identified piRNA targeting mRNAs, FP counts the number of random paired piRNA–mRNA sequences that were mistakenly recognized as piRNA target sites, TN reveals the number of random piRNA–mRNA pairs successfully ruled out by the method, and FN shows the number of overlooked piRNA target mRNA sites. The performance information can be summarized through the receiver operating characteristic (ROC) curve [24]. The ROC curve is a visualization graph that plots (1 − specificity) versus sensitivity when the decision boundary is moved. The better the discriminatory power of the evaluated model is, the more upper-left the resulting ROC curve is, indicating that the model can have high sensitivity even if the false positive rate (FPR, Eq. 5) is controlled to be low. FPR is defined as follows:

5 $$\begin{aligned} \mathrm {1 - Specificity} = \frac{\mathrm {FP}}{ \mathrm {TN} + \mathrm {FP} } = \mathrm {FPR} \end{aligned}$$

The ROC curve property can be summarized by the area-under-curve (AUC) metric. The higher AUC is, the better prediction performance the model can achieve.

### Performance of the devised deep learning network in piRNA binding target identification

**Fig. 4:**
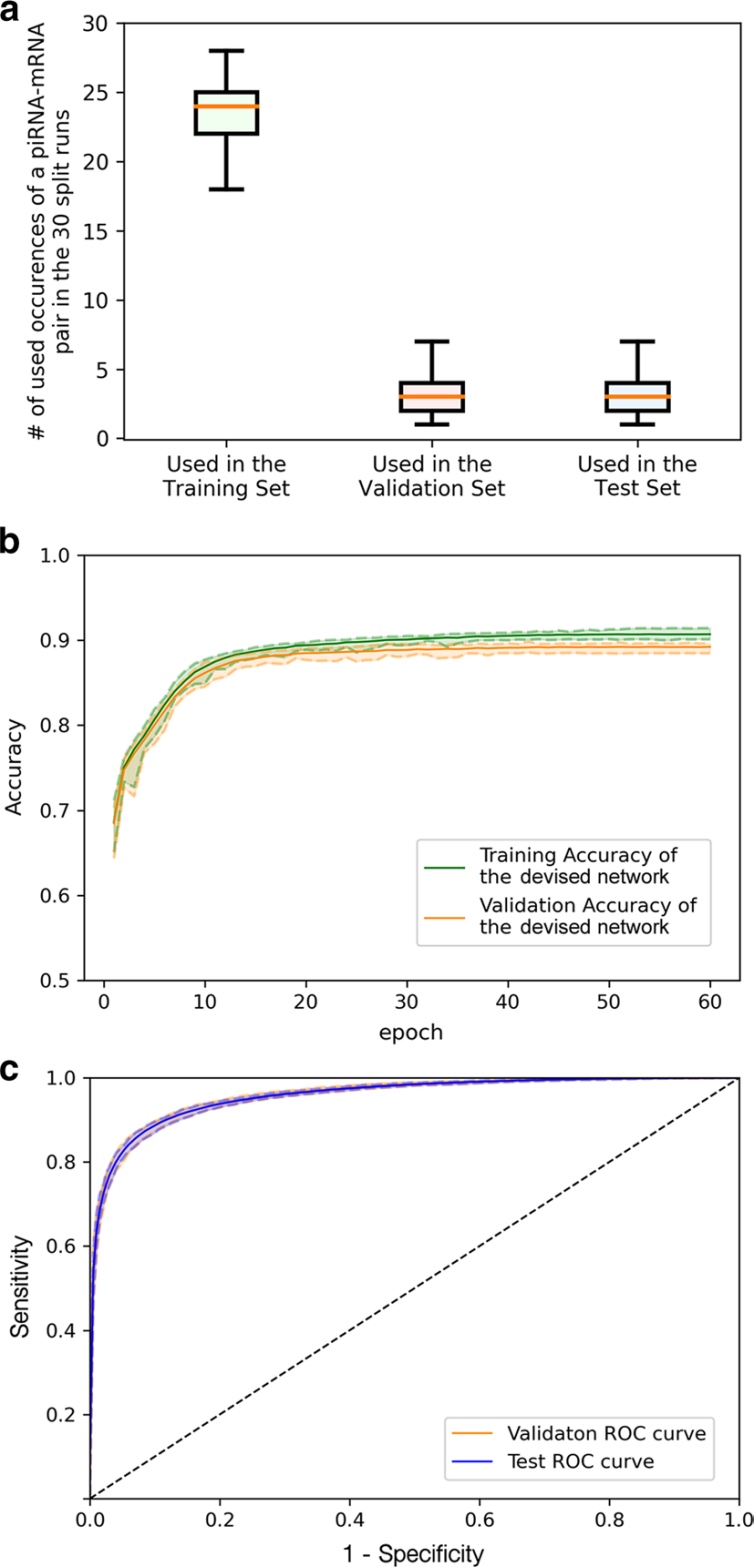
The performance evaluation of the devised network on the wild-type CLASH dataset. The solid green/orange/blue line represents the mean results on the training/validation/test sets in the 30 split runs. And the colored dashed line depicts the variation of the results in the 30 split runs. **a** The randomness of the 30 ten-fold splits was ensured by the average 24/3/3 selected times of each pair to be used in the training/validation/test sets. **b** Learning curves for our devised model. **c** The ROC curve comparison of the validation results and the test results of the devised model

In the standard model learning theory, a prediction model is trained on a training set and the hyperparameters that best suit the model are selected on a different validation set. And in the last, the performance of the trained model is evaluated on an untouched test set. In this research, we applied the ten-fold random splitting techniques to the gathered positive and the negative wild-type CLASH-identified piRNA–mRNA pairs. We randomly split the positive and negative sets into ten folds for 30 times. Randomness is enforced and confirmed in each of 10 fold splitting to avoid biased performance evaluation. In Fig. 4a, the distributions of the number of occurrences of each piRNA–mRNA pair found in the training/validation/test sets are summarized. Among the 30 repeated random splits of positive and negative sets, on average every piRNA–mRNA pair were selected to be included in the training/validation/test sets for 24/3/3 times, reflecting the 8:1:1 random splitting. Hence the randomness of the training/validation/test splits for the 30 runs is ensured. In each time of splitting, eight folds of the datasets were used as the training set, one fold was used as the validation set, and the last fold was adopted as the test evaluation set. We repeated the training process for the 30 splits and obtained the average ROC curves for the validation sets and test sets. The learning curve of the devised model can be checked in Fig. 4b, and the ROC curve results can be found in Fig. 4c. In the learning curve, the training epochs and validation epochs both converge to their prediction accuracy plateaus, showing model convergence on parameters and hyperparameters. Further, the gap between the training and validation curves is substantially small, showing that the hyperparameters do not lead to over-fitting or under-fitting. The final average performance achievements of the devised model on the test sets are as follows: precision = 0.886, recall = 0.899, and F1 = 0.891. And as shown in Fig. 4c, the average test AUC result (AUC = 95.7%) is similar to the average validation result (AUC = 95.8%), and the average ROC curve on the validation sets closely track the ROC curve on the test sets. The AUC and ROC comparison indicated that the hyperparameters were optimally selected in the validation sets and the model performance generalizes well to unknown piRNA–mRNA test pairs. These results prove that the devised model is well-trained and can distinguish true piRNA targeting mRNA sites from random piRNA–mRNA pairs.

### The attention sub-network improves piRNA binding target identification

**Fig. 5:**
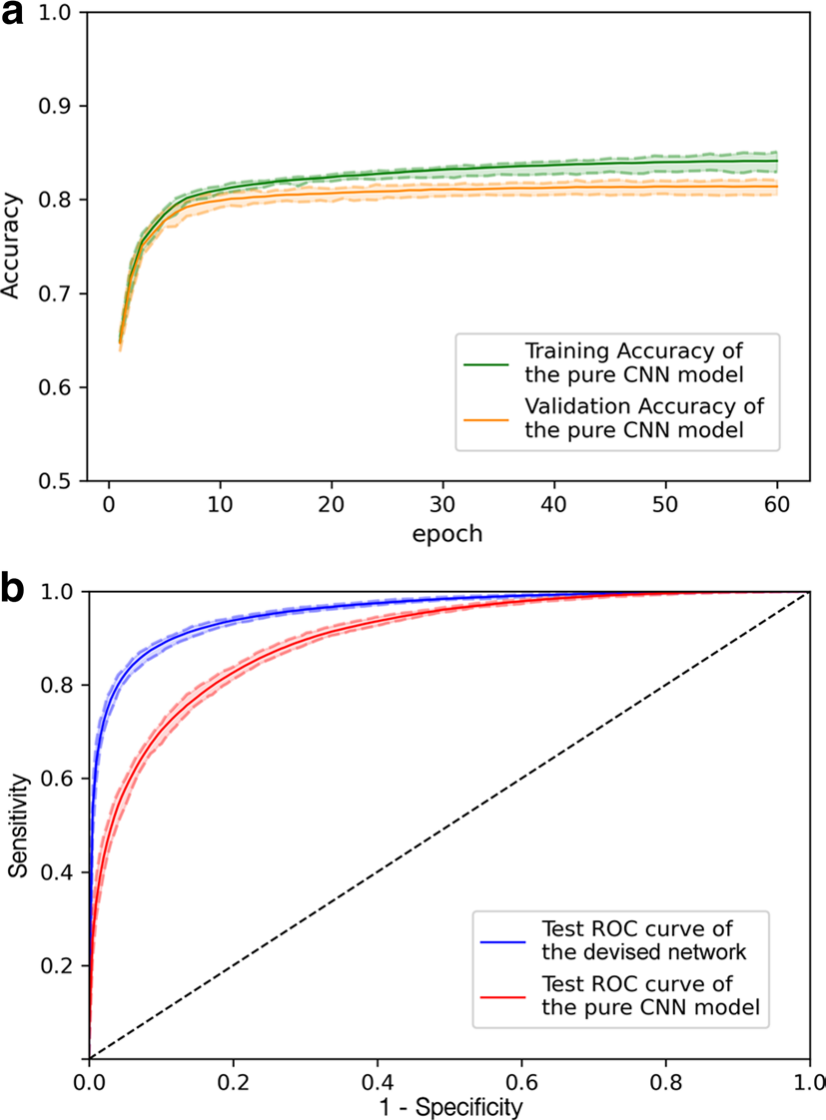
The performance comparison of the devised model and the pure CNN model on the wild-type CLASH ground truth split runs. **a** Learning curves for the pure CNN model. **b** The comparison between the devised network and the pure CNN model on the test sets in the 30 random split runs. The blue line and red line represent the average results of the devised network and the pure CNN model, respectively. And the colored dashed line describes the variation for the models in the 30 split runs

In this research, the designed deep learning architecture utilizes the multi-head attention operation to assay the site-by-site motif patterns of the mRNA segments that match some piRNA sequence binding rule. We compared the devised method to the pure convolutional neural network (CNN) model. The pure CNN model was built based on the same network architecture but with the multi-head attention operation removed. We first trained both models to converge (Fig. 5a) and selected the optimal hyperparameters through the validation sets of the 30 random split runs. The evaluation results of both models were then computed and presented in a ROC curve plot (see Fig. 5b). The learning curve in Fig. 5a ensured that the training process of the pure CNN model was also well and fairly performed. And from the ROC curve results, the devised model that utilizes multi-head attention demonstrated 6.1% (95.7–89.6%, *p* value $=$ 2.87e−11$^*$ by the one-tailed rank-sum test) AUC improvement over the pure CNN model on the test runs. The one-tailed rank-sum test calculates the *p* value against the null hypothesis that the median AUC value of the devised model equals that of the pure CNN model on the 30 test runs. Other performance metric comparisons between the devised method and the pure CNN model on the test runs split from the wild-type CLASH data can be found in Table 2. Overall, the designed multi-head attention operation that concurrently matches the site-by-site motif patterns between piRNA and mRNA sequences can significantly boost the power of distinguishing actual piRNA–mRNA binding sites from random association pairs.

**Table 2:** The performance summary for the devised network and the pure CNN model on the wild-type randomly split test sets

Algorithm model	F1	Precision	Recall
The devised network	0.891* ± 0.003	0.886* ± 0.006	0.899* ± 0.006
The pure CNN model	0.816 ± 0.003	0.805 ± 0.006	0.828 ± 0.005

*indicates that the devised model outperformed the pure CNN model statistically significantly ($\alpha$ = 0.05) using the one-tailed rank-sum test

### The devised deep learning network can be generalized to other independent conditions

We evaluated the devised deep learning network on another independent test set of piRNA targeting mRNA sites. Transcriptome-wide CLASH experiments immunoprecipitated on *C. elegans* PIWI Argonaute PRG-1 were recently performed [16]. In the work of Shen et al., they performed both the wild-type CLASH experiments and the CSR-1 depleted CLASH experiments. As described in the “Methods and Datasets” section, we have merged the two replicates of the wild-type CLASH runs and used them for model training, validation, and preliminary test. We further collected the CSR-1 depleted CLASH runs as an independent test set for model performance and generalization evaluation. Biologists have found out that in young adult worms CSR-1 functions upstream of PRG-1 and piRNA targeting. The protein CSR-1 prevents piRNA binding and protects its mRNA targets from piRNA/PRG1 induced gene silencing [25, 26]. This finding implies that some of the piRNA targeting mRNA binding sites can be occupied by the CSR-1 protein and remains undetected in the wild-type datasets. Thus, when CSR-1 depletion is introduced to the cells, the number of probed unique piRNA targeting mRNA sites can be found to significantly increase and some of extra unknown piRNA–mRNA binding sites can be observed [16]. These additional unobserved piRNA–mRNA binding sites serve to be a candidate independent test set for piRNA–mRNA binding site prediction algorithms.

We merged the two CSR-1 depleted CLASH experimental replicates as an independent dataset. The same procedures for preparing the wild-type positive and negative sets were applied on this independent data. But the piRNA–mRNA binding pairs that were also observed in the wild-type ground truth datasets were removed from the positive set of this independent data. This extra guard guaranteed that the independent positive test set contains no easy case and can help assess the model performance generalization in an unbiased way. We further checked if any of the piRNA targeting mRNA sites in the CSR-1 depleted samples were accidentally included in the negative set generated from the wild-type CLASH runs. No binding pairs observed in CSR-1 depleted CLASH experiments were included in the negative set generated from the wild-type PRG-1 CLASH experiments. This observation ensures that the model was not mistakenly trained on false-negative samples. Finally, to collect the independent test set for model evaluation, we randomly chose 10,000 piRNA–mRNA pairs from the CSR-1 depleted CLASH positive set as the independent positive set and picked 10,000 random piRNA–mRNA pairs from the CSR-1 depleted CLASH negative set as the independent negative set.

**Fig. 6:**
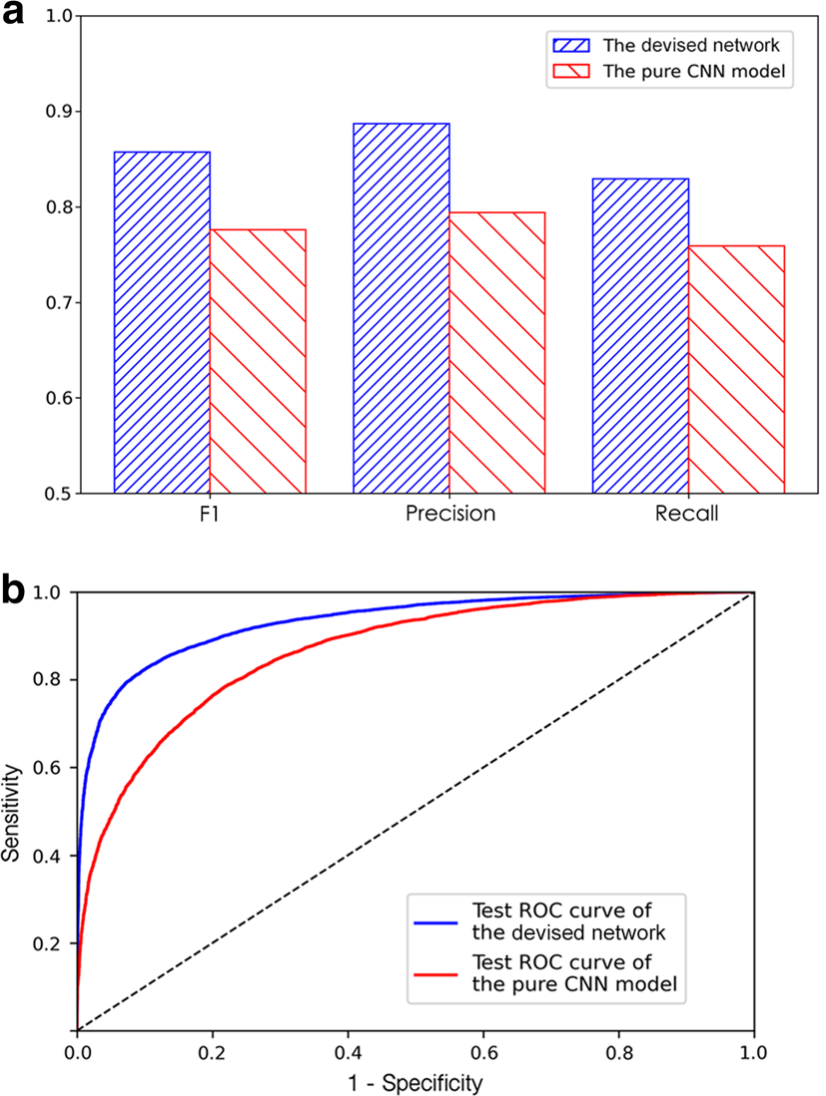
The performance comparison of the devised network and the pure CNN model on the CSR-1 depleted CLASH piRNA–mRNA independent test set. **a** The precision, recall, and F1 value summary of model performance on the independent test set. **b** The ROC results on the independent piRNA–mRNA test set. The blue line represents the result of the devised network, and the red line shows the result of the pure CNN model

We evaluated the performance of the devised network in this independent test set to see if it can be generalized to identify those extra positive piRNA targeting mRNA sites that are usually non-observable in the original CLASH runs of wild-type cells. In this independent test, we assumed that the piRNA binding rules remain unchanged when CSR-1 is deleted [16]. The devised method obtained an AUC of 93.3% in this independent test set (F1 $=$ 0.857, precision = 0.887, recall = 0.830. See Fig. 6a), indicating that our algorithm can successfully extract true piRNA binding targets from genome regions. And the pure CNN model attained lower performance of AUC = 86.6% in this independent test set (F1 $=$ 0.776, precision $=$ 0.794, recall $=$ 0.758). Compared with the pure CNN model built without the multi-head attention operation, the devised multi-head attentive deep network acquired 6.7% (93.3–86.6%, Fig. 6b) improvement in AUC on the independent test set. The independent test comparison results confirm that the way we designed the model by site-by-site probing the binding rules between piRNA and mRNA sequences through multi-head attention is vital for identifying genomic piRNA targeting mRNA binding sites.

## Discussions

### The multi-head attention operation can reveal testable piRNA binding rules

**Fig. 7:**
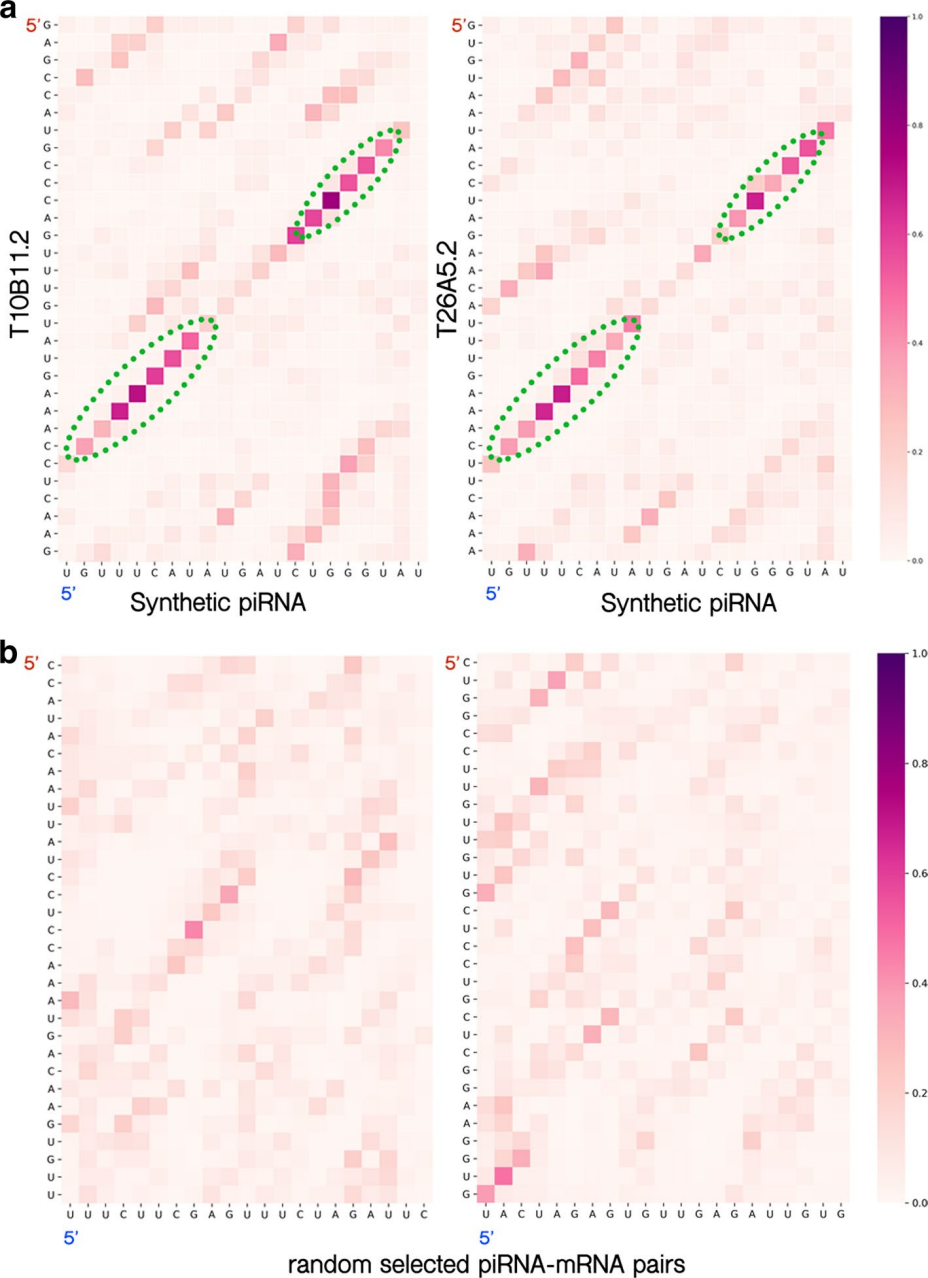
Visualization of the binding rules mined out by the multi-head attention operation. **a** The attention weight matrix visualization for a synthetic piRNA 5’-UGUUUCAUAUGAUCUGGGUAU and its targeting mRNA T10B11.2 and T26A5.2. For cellular piRNA–mRNA binding events, some piRNA binding rule is satisfied. In this case, two seed regions (verified in [10]) are observed. **b** For randomly assigned piRNA–mRNA segment pair, no binding rule can be found in the attention weight matrix visualization

In this research, we have incorporated a multi-head attention operation for piRNA–mRNA binding motif pattern probing to boost the network performance. It is long blamed that deep learning provides merely a black box modeling that is hard to be interpreted. Yet these models have been proven to help extract novel patterns from the data [27]. It is worthy of further investigation of the devised model to understand the logic and details of the deep learning network. In the devised network, the multi-head attention operation is designed to mimic the real piRNA binding rules that can be observed in *C. elegans*. The attention vector designed to be used in Part II of the model (multi-head attentive binding recognition sub-network) can be interpreted as the binding preference of the given piRNA for its targeting mRNA sequences. We take a synthetic *C. elegans* piRNA with sequence 5’-UGUUUCAUAUGAUCUGGGUAU and its target mRNA T10B11.2 and T26A5.2 as our examples. In the devised network, the synthetic piRNA and these two transcripts are identified as true piRNA binding events. The attention vector between the piRNA and its targeting mRNA segment of T10B11.2 and T26A5.2 are visualized in Fig. 7a. As revealed by the attention weight matrix, high weight values were identified on two consensus regions: the 2nd–8th bps and the 15th–19th bps. The higher weight values on these locations indicated extra importance on the piRNA binding features and mRNA motif features considered in these sites. And these sites positively determine the piRNA–mRNA binding events and form the latent piRNA binding rules. In previous researches, this binding rule for the synthetic piRNA has been experimentally inferred and verified [10, 16]. That is, the devised model can identify the real cellular piRNA binding rules and provide testable hypotheses. In contrast to positive piRNA–mRNA binding events, the randomly paired piRNA and the mRNA segment convey random mosaic signals in the attention weight matrix (see Fig. 7b). These random mosaic signals make the classification sub-network in the devised network eliminate the random pair. These examples show that our model not only provides superior identification performance but also extracts the hidden biological piRNA binding preference in cells. These rules can suggest piRNA functional mechanism hypotheses for further subsequent experiments.

### The devised deep learning model outperforms simple baseline score methods

**Fig. 8:**
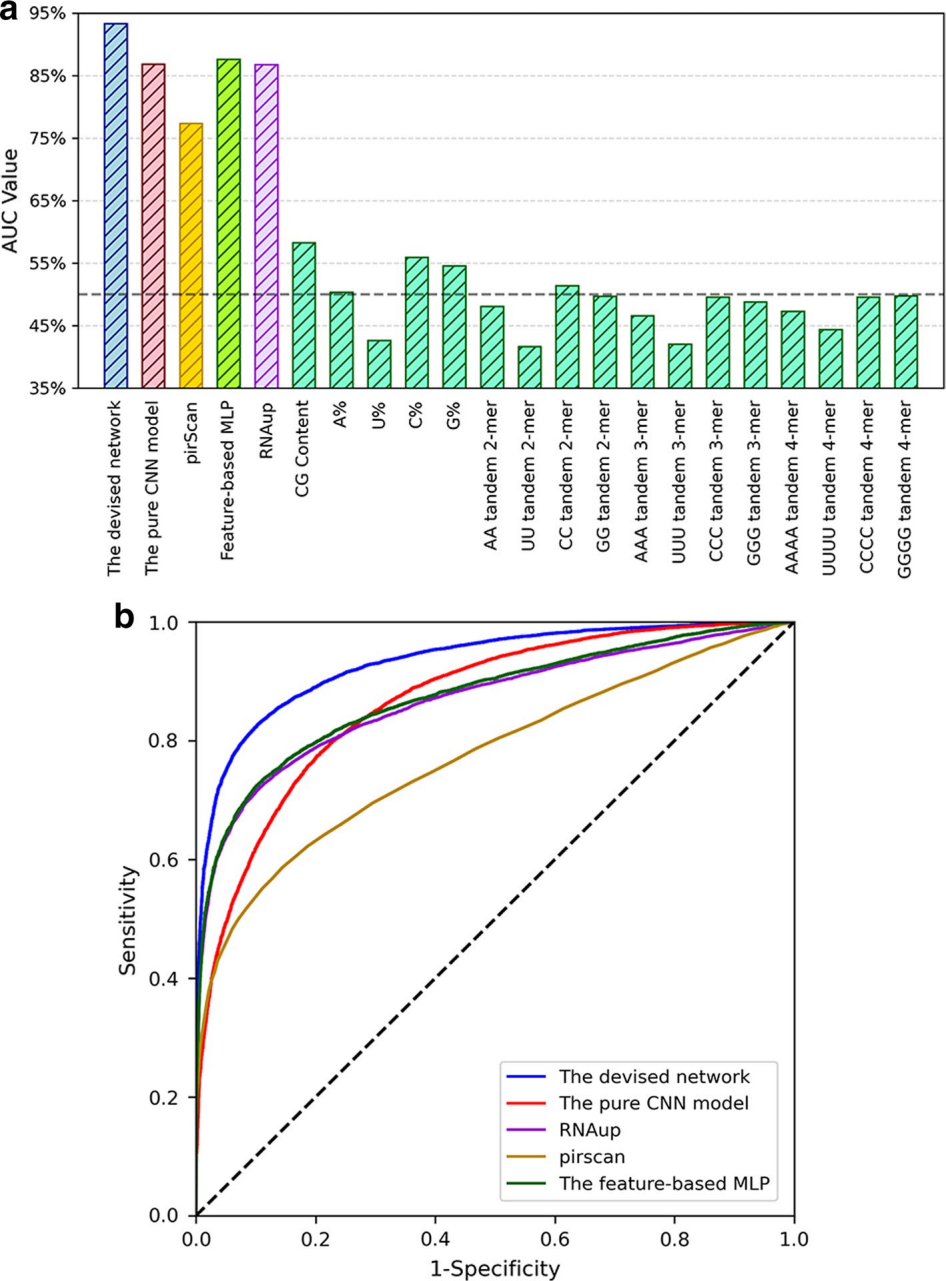
The comparison of the devised model with some baseline score methods on the CSR-1 depleted independent test set. **a** The AUC values of the devised deep learning model, the pure CNN model, RNAup, pirScan, and other 17 baseline composition score methods. A%/U%/C%/G% stand for the nucleotide composition of A/U/C/G, respectively. AUC $=$ 50% represents the random guess performance. **b** The ROC curves of the devised model, the pure CNN model, pirScan, RNAup, and the feature-based MLP model based on binding energy and the 17 nucleotide score features

In the previous section, we have demonstrated that the multi-head attention operation can reveal testable piRNA binding rules in cells. We next show that straightforward search on only one pattern and other nucleotide statistics of mRNAs are not powerful enough to find all real piRNA–mRNA binding pairs. Researchers have developed a pattern search tool called pirScan [11] for rule-based piRNA binding target identification in *C. elegans*. In pirScan, the two regions (seed region 2–7 nts and non-seed region 8–21 nts) of piRNAs are used as the binding patterns to scan through the given mRNA for identifying piRNA–mRNA binding events. A final piRNA targeting score is computed by pirScan for the piRNA–mRNA pair based on the GU and non-GU mismatches in the seed and non-seed regions. We compared the devised model with the results of pirScan to demonstrate the improvement of the devised deep learning model. We also computed 18 primary mRNA whole-sequence statistical features proposed by previous studies as the piRNA–mRNA binding classification baselines: the CG contents of the mRNA fragments, the nucleotide compositions of the mRNAs, k-mer (k = 2, 3, 4) tandem repeats of the mRNAs (features proposed by [15]), and the piRNA–mRNA binding energy calculated by RNAup [22]. These nucleotide statistics convey the basic information of the overall sequence composition. We utilized the ROC curve technique to compare the AUC value of the devised model with the results of pirScan and these 18 baseline statistical scores on the CSR-1 depleted CLASH independent test set. As shown in Fig. 8a, the devised multi-head attention model (AUC = 93.3%) outperforms pirScan (AUC = 77.3%), revealing that pattern search using limited rules can obtain only a fraction of true piRNA–mRNA binding pairs. And the devised model also shows better performance than the RNAup energy score (AUC = 86.7%) and achieves at least 35% better AUC results than the 17 basic nucleotide composition scores. Similar to parts of the feature-based model proposed by Yuan et al. [15], we further trained a multi-layer perceptron (MLP) model based on binding energy and these 17 nucleotide composition feature scores to confirm the effect of the combination of these features. The hyperparameters of the feature-based MLP model were selected using 5-fold cross-validation and random search approaches [28] on the wild-type CLASH dataset (Best model: 3 layers with 128, 128, and 128 nodes, respectively. Dropout = 0.1. Learning rate = 0.00026). From the ROC curves shown in Fig. 8b, the MLP model based on these 18 features (AUC = 87.5%) is still 5.8% lower than the devised model (93.3%) in the AUC performance on the CSR-1 depleted CLASH independent test set. Hence, the devised deep end-to-end attention model can capture more decent site-by-site binding rules and better identify piRNA–mRNA binding pairs than these baseline features.

### The attention mechanism is robust against the lengths of candidate mRNA segment sequences

**Fig. 9:**
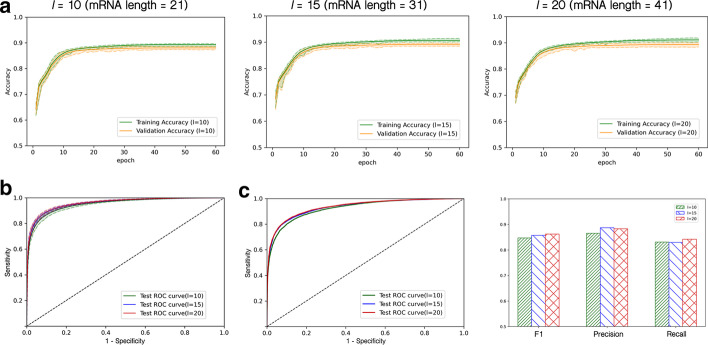
The devised deep learning network is robust against different extended mRNA lengths (*l*). We trained the devised model architecture against different mRNA lengths (21/31/41 bps) on the ground truth dataset and tested them on the independent test set. The input of different mRNA lengths does not affect the performance of the devised model. **a** Learning curves for the model with $l=10, 15, 20$ (corresponding to mRNA sequence length of 21 bps, 31 bps, and 41 bps). **b** The ROC comparison of models with different *l* on the 30 split test sets from the wild-type CLASH data. **c** The ROC curve and performance comparison of models with different *l* on the independent test set

In the protocol of CLASH experiments, the cell samples were treated with RNase to trim the RNA sequences that were not bound by the *C. elegans* PIWI Argonaute PRG-1. Due to RNase treatment, RNA sequences around PRG-1 might be potentially degraded, causing the vague identification of piRNA targeting mRNA sequence segments. Our devised network used the center sites of the mapped start and end transcript locations of a given mRNA segment as the piRNA target site. And to recover the RNA degradation, 15 bps (*l* = 15) were extended upstream and downstream of the center site to form the final binding mRNA sequences. To evaluate the impact of the extended degradation recovering sequence length (*l*), we further tested if the model performance varies with the recovering length. We trained the model on *l* = 10, 15, 20 with total mRNA sequence lengths of 21 bps, 31 bps (the default chosen model), and 41 bps. Then we evaluated the model performance of the devised deep multi-head attention architecture with different extended degradation recovering lengths (*l* = 10, 15, 20) on both the 30 split test sets and the CSR-1 depletion independent test set. The learning curve technique ensured training convergence and optimal hyperparameter selection of models for different mRNA sequence input lengths (see Fig. 9a). The training convergence and the gap between training and validation episodes were controlled by selected best hyperparameters for each architecture with different *l*’s. On the wild-type CLASH ground truth data split test runs, the architecture of *l* = 10 (mRNA length 21 bps) showed slightly lower AUC performance (AUC = 93.9%) than the other two architectures. And the performance of model architectures with *l* = 15 (mRNA length 31 bps, AUC = 95.7%) and with *l* = 20 (mRNA length 41 bps, AUC = 95.8%) resembled each other (see Fig. 9b). Furthermore, the AUC/F1/precision/recall results on the CSR-1 depletion independent test set are collected in Fig. 9c. Similar performance was observed between the model architectures with *l* = 15 (AUC = 93.3%) and with *l* = 20 (AUC = 93.5%). And slightly performance degradation in AUC (AUC = 92.4%) was found in the model architecture with *l* = 10. The F1 comparison for different degradation length *l* also showed around 1% degradation in the model architecture with *l* = 10 and is comparable between the model architectures with *l* = 15 and with *l* = 20. The slight performance deterioration of the mRNA length of 21 bps (*l* = 10) resulted from the incomplete recovery from the RNase treatment effect. These results conclude that the length of 31 base pairs for mRNA sequences can successfully overcome the RNA degradation problem resulted from RNase treatment and was selected to be used in our devised model. Moreover, our model is robust against the extended degradation recovering sequence length *l* as long as sufficient lengths of mRNA sequence segments are considered.

## Conclusions

In this research, we developed the first deep learning architecture to identify piRNA targeting sites on *C. elegans* mRNAs. Besides motif extraction using convolution and squeezing-extraction operation, we also designed a multi-head attention sub-network that helps extract the piRNA binding rules to identify viable piRNA target sequences. Using the technique of random repeated 8:1:1 training/validation/test set splitting, we optimized the devised deep network via the training folds and tuned the hyperparameters on the validation folds. A preliminary performance evaluation was conducted on the test folds. We repeated 30 runs of random training/validation/test splits and showed that on average the optimized novel deep network generalizes well from validation folds to test folds and can achieve supreme average test performance of AUC = 95.7%. Then we showed that the designed multi-head attention sub-network could provide an additional performance improvement over the pure CNN network structure. We also collected an independent test set from the CSR-1 depletion CLASH experiments. Our model made a performance accomplishment of AUC = 93.3% on this independent set and outperformed the pure CNN model over 6.7%. In the last, we showed that a verified binding rule of a synthetic piRNA can be automatically extracted by our model and fit well with the experimental observations. These results demonstrated that the devised model not only has high piRNA–mRNA identification performance but can also suggest testable biological piRNA binding rules for future research. The piRNA–mRNA binding identification deep learning network will be further extended to different animal species when more comprehensive CLASH datasets in these species are available.

## Methods and datasets

### Wild-type CLASH piRNA target site dataset

The cross-linking, ligation, and sequencing of hybrids (CLASH) technique has been developed and used to probe in vivo RNA-RNA interactions [20, 29]. Through chimeric molecules formed between small RNA and its mRNA targets, the binding events between them are assayed. And by sequencing the chimeric molecules, the mRNA target sites of piRNAs can be identified. In a recent study, transcriptome-wide CLASH experiments for measuring the interactions between piRNAs and their targeting mRNAs were performed on the *C. elegans* PIWI Argonaute PRG-1 [16]. We merged the two wild-type CLASH experimental replicates gathered from [16] as the ground truth CLASH data for training and validation of our model. By using these CLASH identified chimeras, in vivo piRNA–mRNA binding events can be extracted. Based on these binding events, we constructed a positive set that contains real rigorous piRNA–mRNA binding pairs and a carefully prepared negative set consisting of verified random-associated piRNA–mRNA pairs. A strict data preparation procedure for identifying experimentally verified random piRNA–mRNA association pairs was designed and followed to obtain the confident negative set. The overall steps for preparing the positive and the negative piRNA–mRNA pairs are summarized in Fig. 2 and elucidated in the “Overview of the deep learning piRNA binding identification process” section.

### The devised deep learning piRNA binding target identification network

The designed deep learning network for piRNA–mRNA binding identification can be divided into three sub-networks: the motif feature extraction sub-network, the multi-head attentive binding recognition sub-network, and the classification sub-network (see Fig. 1). And the network structure is summarized in Table 1.

#### Part I: motif feature extraction sub-network

The devised deep network takes a piRNA sequence and the potential target mRNA sequence segment as its inputs. First, the given piRNA and mRNA segment sequences are transformed into one-hot encoding for the four nucleotides (A, U, G, C). The transformation results in a 21 by 4 encoding vector for the given piRNA sequence and a 31 by 4 encoding vector for the corresponding mRNA sequence segment. Each vector is then fed into an individual convolution operation followed by one extra squeezing-and-excitation block (SE block, see Fig. 1-Part I). The convolution operator described in Eq. 6 serves to extract significant sequence motifs from the two encoding vectors:

6 $$\begin{aligned} C_{n} = E \circledast K_n \mathrm {, \ where\ } C_{n} (j) = \sum _{q=j-m}^{j+m} \sum _{r=1}^{4} K_{n} (q-j+m+1, r) * E(q, r) \end{aligned}$$

In Eq. 6, *E* is the 21 by 4 feature vector for the piRNA or the 31 by 4 feature vector for the mRNA segment from one piRNA–mRNA pair, $C_{n}(j)$ is the *j*th element of the output vector $C_{n}$, $\circledast$ represents the 1D convolution operator, $K_n$ is the *n*th 1 by $(2m+1)$ kernel filter with depth 4, *r* iterates through the four ribonucleotides, and *q* loops over the kernel window. SE blocks described by Eq. 7 are added to further apply layer weighting to the extracted patterns from the previous convolution layer [30]:

7 $$\begin{aligned} s(n) = \frac{1}{E_{len}} \sum _{q=1}^{E_{len}} C_{n}(q) \mathrm {\ and \ } F = a_2(a_1(s W_{S, 1}^T) W_{S, 2}^T)\circ C, \end{aligned}$$

where $E_{len}$ is the length of the input sequence (21 for piRNA sequences and 31 for mRNA sequences), *s* is a 1 by 128 squeezing vector, $W_{S,1}$/$W_{S,2}$ are the trainable weight parameter matrices with a reduction factor of 4, $a_1$ is the ReLU activation function, $a_2$ is the sigmoid activation function, and $\circ$ is the Hadamard product between two matrices. This excitation operation can help the network to focus on more expressive motif patterns identified from the features of the piRNA sequence and the mRNA sequence segment. Detail hyperparameters of the convolution layers and the SE blocks are summarized in Table 1-Part I. After the motif feature extraction sub-network, the given piRNA sequence and the mRNA sequence segment are transformed into two pattern feature matrices for the next stage of the multi-head attentive binding recognition sub-network. We denote the resulting feature matrix *F* as *P* for piRNA and as *M* for the mRNA sequence segment in the following subsections.

#### Part II: multi-head attentive binding recognition sub-network

To simultaneously consider the binding features of the given piRNA sequence and the motif features of the given mRNA sequence segments, we designed a multi-head attention sub-network to recognize piRNA binding rules. The attention operation was first proposed in natural language processing to help the neural machines focus on the relative importance of words in a sentence to every other word [31]. Following this concept, we designed a site-by-site multi-head attention operation for calculating the binding motif dominance in the given piRNA–mRNA pair. The extracted feature at site *i* of the mRNA sequence segment is attended with each of the site features of the given piRNA sequence as in Eq. 8:

8 $$\begin{aligned} H_{t} = \mathrm {softmax}\,\left(\frac{ (MW_{Q, t}) (PW_{K,t})^T}{\sqrt{d}}\right)\, (PW_{V,t}), \end{aligned}$$

where $H_{t}$ is the *t*th attended result matrix, *P* is the extracted motif feature matrix for the given piRNA, *M* is the extracted motif feature matrix for the given mRNA sequence segment, *d* is the sequence length of piRNA (21 in this research), and $W_{Q, t}$/$W_{K,t}$/$W_{V,t}$ are the trainable multi-head dimension transformation weight parameter matrices. The attention operation in Eq. 8 computes the dominance importance of the binding feature of every site of the given piRNA sequence to site *i* in the mRNA sequence. Then the dominance importance values of mRNA site *i* to every piRNA site motif feature are used as the attention weighting to sum over the piRNA motif features for estimating piRNA binding affinity. This operation is repeated for every site of the 31 nts in the mRNA sequence segment. To allow diverse motif feature consideration, we further added the multi-head techniques to the site-by-site attention operation. Diverse attention operations are parallelly incorporated in the network to consider different representation spaces of the piRNA–mRNA binding features. The designed multi-head attention operation is shown in Eq. 9:

9 $$\begin{aligned} H = (H_1 \oplus H_2 \oplus \ldots \oplus H_k) W_H^T, \end{aligned}$$

where $\oplus$ is the concatenation operation that lines up the vectors, $W_H$ is the trainable weight parameter matrix that summarizes the attended motifs from different heads, and *H* is the final multi-head attention vector.

A residual feed-forward fully connected layer is added to facilitate the training process. In addition to normal fully connected multi-layers, the input vector of the fully connected layer is forwarded to be summed up with the output of the fully connected layer (Eq. 10).

10 $$\begin{aligned} K = H + M, \ R = a(a(K W_{R, 1}^T) W_{R, 2}^T) + K, \end{aligned}$$

where *H* is the multi-head attention vector, *a*(.) represents the activation function (in which PReLU is used), and $W_{R,1}$/$W_{R,2}$ are the trainable weight parameter matrices in the fully connected sub-layers. Following this residual network construct, the model can converge more easily in the training process and thus provide extra performance boost [32]. Dropout and layer normalization were added after the multi-head attention layer and within the residual feed-forward fully connected layer to enhance the model performance. The detailed sub-network architecture can be found in Table 1-Part II.

#### Part III: classification sub-network

In the last part of the network, the attentive feature vectors of the piRNA and its potential targeting mRNA segments generated by the multi-head attention network are used for identifying real piRNA silencing targets. In this classification sub-network, two layers of fully connected neural nets (Eq. 11) are stacked to mapped the attentive features into a feature-separable high dimensional space wherein functional piRNA–mRNA cellular binding events and randomly matched pairs are distinguishable:

11 $$\begin{aligned} p = \mathrm {softmax}(a(a(R_{\mathrm {flatten}} W_{C, 1}^T) W_{C, 2}^T)), \end{aligned}$$

where *p* is the final probability that the piRNA binds to the given mRNA sequence segment, $R_{\mathrm {flatten}}$ is the flattened 1 by (31 * 128) vector of *R*, *a*(.) represents the activation function (in which PReLU is used), softmax(.) is the operation that turns a vector into a probability mass distribution, and $W_{C, 1}$/$W_{C, 2}$ are the trainable parameter matrices. Dropout and batch-norm layers were added to each fully connected neural layer to boost the model performance and generalization for unseen data. We summarized the structure of the binding classification sub-network in Table 1-Part III. After the binding classification sub-network, the binding event of the given piRNA to the mRNA segments is identified.

## Data Availability

The positive and the negative sets from the wild-type CLASH data, the positive and the negative independent test sets from the CSR-1 depleted CLASH data, and the devised deep multi-head attention network model codes can be downloaded freely online from http://cosbi2.ee.ncku.edu.tw/data_download/piRNA_mRNA_binding.

## Abbreviations

piRNA: Piwi-interacting RNA
ncRNA: Non-coding RNA
CLIP: Cross-linking immunoprecipitation
CLASH: Cross-linking, ligation, and sequencing of hybrids
AUC: Area under the curve
LBS: Loose piRNA–mRNA binding pair set
NCS: Negative candidate set
SE block: Squeezing-and-excitation block
ROC curve: Receiver operating characteristic curve
FPR: False positive rate
CNN: Convolutional neural network
MLP: Multi-layer perceptron
